# Molecular characterization of Brazilian wild-type strains of bovine respiratory syncytial virus reveals genetic diversity and a putative new subgroup of the virus

**DOI:** 10.1080/01652176.2020.1733704

**Published:** 2020-03-05

**Authors:** Raquel Arruda Leme, Alais Maria Dall Agnol, Luciana Carvalho Balbo, Fernanda Louise Pereira, Flávia Possatti, Alice Fernandes Alfieri, Amauri Alcindo Alfieri

**Affiliations:** a National Institute of Science and Technology of Dairy Production Chain (INCT-Leite), Universidade Estadual de Londrina, Paraná, Brazil; b Laboratory of Animal Virology, Department of Veterinary Preventive Medicine, Universidade Estadual de Londrina, Londrina, Paraná, Brazil

**Keywords:** Bovine, bovine orthopneumovirus, bovine respiratory disease, BRD, BRSV, calf

## Abstract

**Background:**

Bovine orthopneumovirus, formerly known as bovine respiratory syncytial virus (BRSV), is frequently associated with bovine respiratory disease (BRD).

**Aim:**

To perform the molecular characterization of the G and F proteins of Brazilian wild-type BRSV strains derived from bovine respiratory infections in both beef and dairy cattle.

**Materials and Methods:**

Ten BRSV strains derived from a dairy heifer rearing unit (*n* = 3) in 2011 and steers of three other feedlots (*n* = 7) in 2014 and 2015 were analyzed. For the BRSV G and F partial gene amplifications, RT-nested-PCR assays were performed with sequencing in both directions with forward and reverse primers used.

**Results:**

The G gene-based analysis revealed that two strains were highly similar to the BRSV sequences representative of subgroup III, including the Bayovac vaccine strain. However, the remaining seven Brazilian BRSV strains were diverse when compared with strains representative of the BRSV I to VIII subgroups. The central hydrophobic region of the Brazilian BRSV G gene showed the replacement of conserved cysteines and other residues of importance to antibody reactivity. The deduced F gene amino acid sequences from the Brazilian BRSV strains showed changes that were absent in the representative sequences of the known subgroups. Viral isolation on the nasopharyngeal swab suspensions failed to isolate BRSV.

**Conclusion:**

Results suggest that these strains represent a putative new subgroup of BRSV with mutations observed in the immunodominant region of the G protein. However, further studies on these Brazilian BRSV strains should be performed to establish their pathogenic potential.

## 1. Introduction

Bovine respiratory disease (BRD) is a multifactorial and multietiological syndrome in which management and environmental risk factors, in addition to etiologic agents such as viruses and bacteria, are involved (Gershwin et al., 2015). The main bacterial agents are *Mannheimia haemolytica, Mycoplasma bovis, Pasteurella multocida*, and *Histophilus somni* (Panciera and Confer 2010, Gershwin et al. 2015, Baptista et al. 2017). Bovine respiratory disease virus (BRSV), bovine viral diarrhea virus (BVDV), bovine alphaherpesvirus 1 (BoHV-1), bovine parainfluenza virus 3 (BPIV-3), and bovine coronavirus (BCoV) are the possible viral causes of BRD (Beuttemmuller et al. 2017, Headley et al. 2018). Among these microorganisms, BRSV is frequently associated with BRD (Apley 2006, Grissett et al. 2015).

BRSV infections are widely distributed in cattle herds throughout the world, with occasional annual outbreaks, especially in winter and autumn, in temperate climates (Sarmiento-Silva et al. 2012). The illness is frequently characterized by interstitial pneumonia and bronchiolitis, mainly affecting calves up to one year of age as well as confined and immunologically compromised adult animals in feedlots (Furze et al. 1997, Larsen et al. 2000, Gershwin 2007, Sacco et al. 2014).

Until 2015, BRSV was classified into the *Pneumovirus* genus, *Paramyxoviridae* family (ICTV 2015). However, this classification was revised, and BRSV was renamed bovine orthopneumovirus species and is currently classified into the *Orthopneumovirus* genus within the newly created *Pneumoviridae* family (ICTV 2018). BRSV is an enveloped and pleomorphic virus with a negative-sense RNA genome approximately 15,000 nucleotides in length that encodes 10 proteins. The lipid envelope of BRSV consists of three surface glycoproteins, defined as the attachment glycoprotein (G), fusion (F) protein, and the small hydrophobic (SH) protein (Sarmiento-Silva et al. 2012). The F and G genes play important roles in viral infectivity and are the major targets of the immune system (Larsen et al. 2000, Valarcher and Taylor 2007).

The attachment glycoprotein G is responsible for virus-cell binding, while the F protein is responsible for virus penetration into the cell, the spread of the virus in the host organism, and the formation of the characteristic syncytia (Valentova 2012). According to Valarcher and colleagues (2000), the F and G genes present a genetic variability of 2% and 8%, respectively, revealing that the F protein is highly conserved, especially when compared with the G protein.

The G protein composes the most external viral layer and has three domains: cytoplasmic, located between amino acids (aas) 1-37; transmembrane, between aas 38-65, and extracellular or ectodomain, between aas 66-257 (Valentova 2012). This last domain consists of a highly conserved hydrophobic central region composed of 32 aas and four cysteines that form two disulfide bridges (Doreleijers et al. 1996). Due to its high genetic variation, the G protein may be used for evolutionary analyses of BRSV strains (Valarcher et al. 2000). Based on G gene analysis, BRSV strains were divided into four subgroups designated as A, B, intermediate or AB, and nontyped (Furze et al. 1994, Schrijver et al. 1996, Furze et al. 1997). Following, several studies have focused on the genetic evolution of BRSV on the basis of both G and F sequences and currently the BRSV strains are classified into subgroups I to VIII (Valarcher et al. 2000, Klem et al. 2014, Bertolotti et al. 2018, Krešić et al. 2018).

The aim of this study was to perform the molecular characterization of the G and F proteins of Brazilian wild-type BRSV strains derived from bovine respiratory infections in both beef and dairy cattle.

## 2. Materials and methods

The study was submitted to the Ethics Committee on Animal Experiments of the Universidade Estadual de Londrina and approved under the identification number 1835.2019.45. All applicable international, national, and/or institutional guidelines for the care and use of animals were followed.

The Brazilian BRSV strains in this study were obtained from nasopharyngeal swabs (*n* = 10) that were part of a biological sample collection of the Laboratory of Animal Virology of the State University of Londrina. These strains were derived from four different cattle herds. The first sampled herd was a dairy heifer rearing unit (herein referred to as Me) located in the western region of Paraná state. The second herd (herein referred to as RP) was a beef cattle feedlot from the southern region of São Paulo state. The other two herds were beef cattle feedlots (herein referred to as Or and PG) located in the north and central-west regions of Paraná state. Three strains (UEL01-Me, UEL02-Me, and UEL03-Me) were collected from acute respiratory disease-affected dairy heifers in 2011. Three (BRA-UEL04-RP, BRA-UEL05-RP, and BRA-UEL06-RP) and four (UEL07-Or, UEL08-PG, UEL09-PG, and UEL10-PG) strains were obtained from samples collected in 2014 and 2015, respectively, from 2-year-old steers with clinical signs of respiratory disease, including nasal discharge and pyrexia. None of the four cattle herds had vaccination history against BRSV.

Nucleic acid extraction was performed from 500 μL aliquots of the nasopharyngeal swabs with PBS, which were pretreated with sodium dodecyl sulfate (SDS) and Proteinase K (Ambion^®^ Life Technologies™, Carlsbad, CA, USA) at a final concentration of 1% and 2 mg/mL, respectively, and incubated at 56 °C for 30 min. Then, the silica/guanidinium isothiocyanate extraction method (Boom et al. 1990) was used. The extracted nucleic acid was eluted in 50 μL of ultra-pure RNase-free diethylpyrocarbonate (DEPC)-treated sterile water (Invitrogen™ Life Technologies™, Carlsbad, CA, USA) and stored at −80 °C until analysis. Sterile water aliquots were included as negative controls during nucleic acid extraction and the following procedures.

For the BRSV G and F partial gene amplifications, RT-nested-PCR assays were performed according to Vilček et al. (1994a) and Valarcher et al. (2000), respectively, using the Superscript™ III Reverse Transcriptase (Invitrogen^®^ Life Technologies, Eugene, OR, USA) and the Platinum Taq DNA Polymerase (Invitrogen™ Life Technologies, Carlsbad, CA, USA), according to the manufacturer’s instructions. The expected fragment sizes were 371 bp for the G gene and 833 bp for the F gene. In addition, other bovine respiratory tract pathogens were investigated, including *Mannheimia haemolytica* (Angen et al. 2009), *Pasteurella multocida* (Townsend et al. 1998), *Histophilus somni* (Angen et al. 1998), *Mycoplasma bovis* (Voltarelli et al. 2018), BVDV (Vilček et al. 1994), BoHV-1 (Claus et al. 2005), BCoV (Takiuchi et al. 2006), and BPIV-3 (Zhu et al. 2011). The amplified products were analyzed by 2% agarose gel electrophoresis in TBE buffer, pH 8.4 (89 mM Tris, 89 mM boric acid, and 2 mM EDTA) stained with ethidium bromide (0.5 μg/mL) and visualized under ultraviolet light (UV).

The amplicons were purified using the PureLink^®^ Quick Gel Extraction Kit (Invitrogen, Carlsbad, CA, USA), quantified with a Qubit™ Fluorometer (Invitrogen™ Life Technologies, Eugene, OR, USA), and analyzed by electrophoresis on a 2% agarose gel. The ABI3500 Genetic Analyzer and BigDye™ Terminator v3.1 Cycle Sequencing Kit (Applied Biosystems, Foster City, CA, USA) was used for sequencing, which was performed in both directions with the forward and reverse primers used for nested-PCR for both the BRSV G and F partial genes. Sequence quality analyses were performed using Phred and CAP3 software (http://asparagin.cenargen.embrapa.br/phph/). Similarity searches were performed with sequences deposited in GenBank using Basic Local Alignment Search Tool (BLAST) software (http://blast.ncbi.nlm.nih.gov/Blast.cgi). Phylogenetic tree construction based on nucleotide (nt) sequences were obtained using the maximum-likelihood method and Tamura & Nei model (Tamura and Nei 1993), which provided statistical support with 1,000 bootstrap replicates using the MEGA package (version 7.0) (Kumar et al. 2016). Sequence identity matrixes were performed using BioEdit software version 7.2.5 (http://www.mbio.ncsu.edu/bioedit/bioedit.html). GenBank accession numbers of BRSV strains herein are MK599389 to MK599398 to the G gene and MK599399 to MK599405 to the F gene.

Attempts to isolate the virus in cell culture were performed in Madin Darby bovine kidney (MDBK) and human epithelial type 2 (HEp-2) cells, which were grown in Dulbecco’s Modified Eagle Media (DMEM, Gibco^TM^ BRL, USA), supplemented with 10% fetal calf serum (Gibco^TM^ BRL, USA). Aliquots of 500 µL from nasopharyngeal swab suspensions pre-treated with penicillin, streptomycin, and Gibco Amphotericin B (Gibco^TM^ Antibiotic-Antimycotic, USA) were inoculated on cell cultures following routine procedures by up to four consecutive blind passages. The inoculated MDBK and HEp-2 cells were maintained in CO_2_ incubator (Thermo Electron Corporation^®^, Marietta, Ohio, USA) and inspected daily for cytopathic effect (CPE). The presence of BRSV, BVDV, BoHV-1, BCoV, and BPIV-3 was later investigated by the same RT-PCR procedures previously described.

## 3. Results

All the 10 samples were confirmed as positive for BRSV, showing the expected fragment sizes for the G and F genes. The other respiratory tract pathogens were not amplified in any of the samples.

### 3.1. Gene G analysis

**Figure 1.**
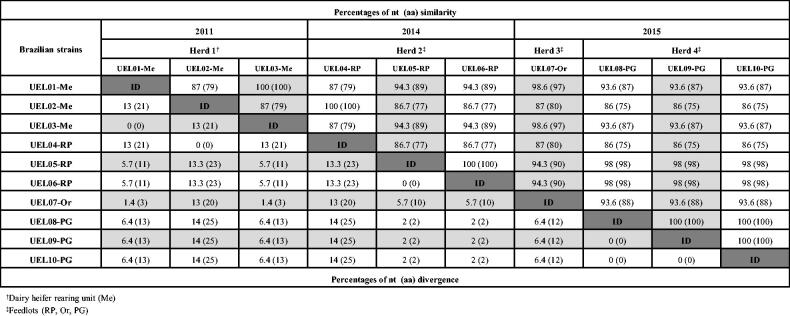
Percentages of similarities and divergences of the nucleotide (nt) and deduced amino acid (aa) sequences among Brazilian bovine respiratory syncytial virus strains in this study based on partial (302 nt) G gene analysis.

The analysis of the partial G gene of the Brazilian BRSV strains revealed that UEL01-Me and 03-Me; UEL02-Me and 04-RP; UEL05-RP and 06-RP; and UEL08-PG, 09-PG, and 10-PG were 100% identical to each other at both the nucleotide (nt) and deduced amino acid (aa) levels. However, the analysis also showed relative diversity among the strains, reaching up to 14% and 25% divergence in the nt and aa sequences, respectively (Figure 1).

The comparison of the nt and aa sequences among the Brazilian strains and reference sequences representative of BRSV I to VIII subgroups revealed similarities in nt sequences that varied from 80 to 89% (70 to 84% for aa sequences). The exceptions were the Brazilian strains UEL02-Me and 04-RP, which showed high similarities (99% and 97-99% at the nt and aa levels, respectively) with BRSV sequences representative of subgroup III (Table 1).

**Table 1.** Percentages of similarities of the nucleotide and deduced amino acid sequences among Brazilian bovine respiratory syncytial virus strains and representative strains of different subgroups based on the partial (302 nt) G gene.

Reference strains (GenBank accession number)	Brazilian strains									
	2011			2014			2015			
	UEL01-Me	UEL02-Me	UEL03-Me	UEL04-RP	UEL05-RP	UEL06-RP	UEL07-Or	UEL08-PG	UEL09-PG	UEL10-PG
**Subgroup I**										
4642 (Y08718)	86 (79)	85 (80)	86 (79)	85 (80)	87 (78)	87 (78)	85 (77)	87 (76)	87 (76)	87 (76)
220-60 (Y11205)	85 (77)	86 (80)	85 (77)	86 (80)	87 (76)	87 (76)	85 (75)	86 (74)	86 (74)	86 (74)
127 (Y08716)	86 (79)	85 (80)	86 (79)	85 (80)	87 (78)	87 (78)	85 (77)	87 (76)	87 (76)	87 (76)
BRSV-25-BR (AY862146)	83 (73)	84 (76)	83 (73)	84 (76)	85 (72)	85 (72)	83 (71)	84 (70)	84 (70)	84 (70)
BRSV-108-BR (AY862147)	83 (74)	84 (77)	83 (74)	84 (77)	85 (73)	85 (73)	83 (72)	84 (71)	84 (71)	84 (71)
**Subgroup II**										
Lelystad (U33539)	88 (83)	93 (88)	88 (83)	93 (88)	89 (82)	89 (82)	88 (84)	88 (80)	88 (80)	88 (80)
37_NL (U55254)	88 (83)	93 (88)	88 (83)	93 (88)	89 (82)	89 (82)	88 (84)	88 (80)	88 (80)	88 (80)
RB54 (L27802)	88 (83)	93 (88)	88 (83)	93 (88)	89 (82)	89 (82)	88 (84)	88 (80)	88 (80)	88 (80)
220-69 (Y08720)	88 (83)	92 (88)	88 (83)	92 (88)	89 (82)	89 (82)	88 (84)	88 (80)	88 (80)	88 (80)
220/69W2 (AF188577)	87 (80)	92 (88)	87 (80)	92 (88)	88 (79)	88 (79)	87 (81)	87 (77)	87 (77)	87 (77)
U55251	87 (80)	91 (88)	87 (80)	91 (88)	88 (79)	88 (79)	87 (81)	87 (77)	87 (77)	87 (77)
U55255	88 (83)	93 (88)	88 (83)	93 (88)	89 (82)	89 (82)	88 (84)	88 (80)	88 (80)	88 (80)
9514923 (AF2488588)	87 (79)	91 (86)	87 (79)	91 (86)	87 (77)	87 (77)	87 (80)	87 (77)	87 (77)	87 (77)
9616957 (AF248590)	86 (81)	91 (88)	86 (81)	91 (88)	88 (80)	88 (80)	86 (82)	87 (78)	87 (78)	87 (78)
9911060-2 (AF248610)	86 (80)	90 (87)	86 (80)	90 (87)	87 (79)	87 (79)	86 (81)	87 (77)	87 (77)	87 (77)
9314893 (U92102)	86 (80)	91 (87)	86 (80)	91 (87)	87 (79)	87 (79)	86 (81)	87 (77)	87 (77)	87 (77)
9402020 (U92103)	87 (80)	91 (87)	87 (80)	91 (87)	88 (78)	88 (78)	87 (81)	87 (76)	87 (76)	87 (76)
9402022 (U92104)	87 (80)	91 (87)	87 (80)	91 (87)	88 (78)	88 (78)	87 (81)	87 (76)	87 (76)	87 (76)
9510237 (U92108)	87 (79)	91 (86)	87 (79)	91 (86)	87 (77)	87 (77)	87 (80)	87 (77)	87 (77)	87 (77)
9510597 (U92109)	87 (81)	91 (87)	87 (81)	91 (87)	88 (80)	88 (80)	87 (82)	87 (78)	87 (78)	87 (78)
9610240 (U92113)	87 (79)	91 (86)	87 (79)	91 (86)	87 (77)	87 (77)	87 (80)	87 (77)	87 (77)	87 (77)
9616348 (U92114)	87 (80)	92 (89)	87 (80)	92 (89)	88 (79)	88 (79)	87 (81)	87 (77)	87 (77)	87 (77)
48036/MA/2018 (LR699780)	86 (78)	90 (86)	86 (78)	90 (86)	86 (76)	86 (76)	86 (79)	85 (74)	85 (74)	85 (74)
48036/MA/2018 (LR669781)	86 (78)	90 (86)	86 (78)	90 (86)	86 (76)	86 (76)	86 (79)	85 (74)	85 (74)	85 (74)
LNP02 (AY507917)	87 (80)	91 (87)	87 (80)	91 (87)	87 (78)	87 (78)	87 (81)	86 (76)	86 (76)	86 (76)
NJ02 (AY507919)	86 (78)	90 (85)	86 (78)	90 (85)	87 (76)	87 (76)	86 (79)	86 (74)	86 (74)	86 (74)
PR03 (AY507921)	86 (80)	91 (87)	86 (80)	91 (87)	87 (78)	87 (78)	86 (81)	86 (76)	86 (76)	86 (76)
SJ02 (AY507922)	86 (79)	91 (86)	86 (79)	91 (86)	87 (77)	87 (77)	86 (80)	86 (75)	86 (75)	86 (75)
ST02 (AY507923)	87 (80)	91 (87)	87 (80)	91 (87)	87 (78)	87 (78)	87 (81)	86 (76)	86 (76)	86 (76)
VN02 (AY507924)	86 (79)	91 (86)	86 (79)	91 (86)	87 (77)	87 (77)	86 (81)	86 (75)	86 (75)	86 (75)
VS97 (AY507925)	84 (75)	88 (80)	84 (75)	88 (80)	85 (76)	85 (76)	84 (76)	85 (74)	85 (74)	85 (74)
9910370 (AF248602)	85 (78)	90 (85)	85 (78)	90 (85)	86 (77)	86 (77)	85 (79)	86 (75)	86 (75)	86 (75)
9910415 (AF248603)	86 (79)	91 (86)	86 (79)	91 (86)	87 (78)	87 (78)	86 (80)	87 (76)	87 (76)	87 (76)
9910670 (AF248608)	86 (78)	90 (85)	86 (78)	90 (85)	87 (77)	87 (77)	86 (79)	86 (75)	86 (75)	86 (75)
MVR553 (U24714)	88 (83)	93 (88)	88 (83)	93 (88)	89 (82)	89 (82)	88 (84)	88 (80)	88 (80)	88 (80)
A3/N/09 (KF501151)	86 (80)	90 (86)	86 (80)	90 (86)	87 (79)	87 (79)	86 (81)	86 (77)	86 (77)	86 (77)
O2/N/10 (KF501153)	85 (78)	90 (85)	85 (78)	90 (85)	87 (78)	87 (78)	85 (79)	86 (77)	86 (77)	86 (77)
Os1/N/10 (KF501154)	86 (79)	90 (86)	86 (79)	90 (86)	87 (79)	87 (79)	86 (80)	86 (76)	86 (76)	86 (76)
O3/N/10 (KF501155)	86 (79)	90 (86)	86 (79)	90 (86)	87 (79)	87 (79)	86 (80)	86 (77)	86 (77)	86 (77)
R1/N/11 (KF501172)	85 (78)	90 (85)	85 (78)	90 (85)	87 (78)	87 (78)	86 (80)	86 (76)	86 (76)	86 (76)
O4-9B/N/11 (KF501169)	86 (80)	90 (86)	86 (80)	90 (86)	87 (79)	87 (79)	86 (81)	86 (77)	86 (77)	86 (77)
F1/0807/SE(JN619441)	87 (80)	91 (86)	87 (80)	91 (86)	87 (77)	87 (77)	87 (81)	86 (75)	86 (75)	86 (75)
H3/0912/SE (JN619444)	86 (80)	91 (87)	86 (80)	91 (87)	87 (78)	87 (78)	86 (81)	86 (76)	86 (76)	86 (76)
U1/0908/SE (JN619451)	87 (80)	91 (87)	87 (80)	91 (87)	87 (78)	87 (78)	87 (81)	86 (76)	86 (76)	86 (76)
W3/0907/SE (JN619455)	87 (80)	91 (87)	87 (80)	91 (87)	87 (78)	87 (78)	87 (81)	86 (76)	86 (76)	86 (76)
**Subgroup III**										
375 (FJ555202)	87 (79)	99 (97)	87 (79)	99 (97)	86 (76)	86 (76)	87 (80)	85 (74)	85 (74)	85 (74)
375 (AF188579)	87 (79)	99 (97)	87 (79)	99 (97)	86 (76)	86 (76)	87 (80)	85 (74)	85 (74)	85 (74)
375_USA (U55256)	87 (80)	99 (99)	87 (80)	99 (99)	86 (78)	86 (78)	87 (81)	86 (76)	86 (76)	86 (76)
391-2 (M58307)	89 (81)	93 (85)	89 (81)	93 (85)	89 (80)	89 (80)	89 (82)	89 (80)	89 (80)	89 (80)
FS-1 (AF188581)	87 (80)	99 (99)	87 (80)	99 (99)	87 (78)	87 (78)	87 (81)	86 (76)	86 (76)	86 (76)
85-1330 (U24716)	89 (83)	92 (87)	89 (83)	92 (87)	88 (82)	88 (82)	89 (84)	88 (80)	88 (80)	88 (80)
ESK/25/TR (MH133326)	84 (78)	87 (80)	84 (78)	87 (80)	85 (79)	85 (79)	84 (79)	85 (77)	85 (77)	85 (77)
ESK/51/TR (MH133327)	84 (78)	87 (80)	84 (78)	87 (80)	85 (79)	85 (79)	84 (79)	85 (77)	85 (77)	85 (77)
YM03 (LC499993)	85 (76)	88 (82)	85 (76)	88 (82)	85 (78)	85 (78)	85 (77)	85 (76)	85 (76)	85 (76)
FKI11 (LC499994)	85 (77)	87 (80)	85 (77)	87 (80)	87 (79)	87 (79)	85 (78)	86 (77)	86 (77)	86 (77)
AK01 (LC499988)	86 (77)	87 (80)	86 (77)	87 (80)	87 (79)	87 (79)	86 (78)	87 (77)	87 (77)	87 (77)
MY01 (LC499989)	85 (77)	89 (82)	85 (77)	89 (82)	86 (79)	86 (79)	85 (78)	85 (77)	85 (77)	85 (77)
IT48170 (KY753469)	83 (69)	89 (79)	83 (69)	89 (79)	83 (69)	83 (69)	83 (70)	84 (71)	84 (71)	84 (71)
USII/S1 (KU159366)	86 (79)	85 (78)	86 (79)	85 (78)	87 (78)	87 (78)	87 (80)	87 (76)	87 (76)	87 (76)
Lehmkhul-Bayovac (L10925)	87 (79)	99 (97)	87 (79)	99 (97)	86 (76)	86 (76)	87 (80)	85 (74)	85 (74)	85 (74)
**Subgroup IV**										
VC464 (AF188582)	89 (83)	95 (91)	89 (83)	95 (91)	89 (81)	89 (81)	89 (84)	88 (79)	88 (79)	88 (79)
Dorset (U24715)	89 (83)	94 (91)	89 (83)	94 (91)	89 (81)	89 (81)	89 (84)	88 (79)	88 (79)	88 (79)
SNOOK (Y08719)	88 (83)	93 (91)	88 (83)	93 (91)	89(81)	89 (81)	88 (84)	88 (79)	88 (79)	88 (79)
WBH (Y08717)	85 (78)	87 (81)	85 (78)	87 (81)	86 (78)	86 (78)	85 (79)	86 (76)	86 (76)	86 (76)
Waiboerhoeve (Y10774)	85 (77)	87 (80)	85 (77)	87 (80)	86 (77)	86 (77)	85 (78)	86 (75)	86 (75)	86 (75)
A51908 (AF295544)	85 (77)	86 (80)	85 (77)	86 (80)	87 (76)	87 (76)	85 (75)	86 (74)	86 (74)	86 (74)
ATCC51908-USA (AF295543)	88 (82)	93 (88)	88 (82)	93 (88)	88 (80)	88 (80)	88 (83)	87 (78)	87 (78)	87 (78)
ATue51908 (AF092942)	88 (82)	93 (88)	88 (82)	93 (88)	88 (80)	88 (80)	88 (83)	87 (78)	87 (78)	87 (78)
9910215(AF248599)	87 (78)	91 (85)	87 (78)	91 (85)	87 (77)	87 (77)	87 (79)	86 (75)	86 (75)	86 (75)
9910501-2 (AF248605)	86 (77)	91 (86)	86 (77)	91 (86)	86 (76)	86 (76)	86 (78)	86 (74)	86(74)	86 (74)
9910639 (AF248607)	87 (78)	91 (85)	87 (78)	91 (85)	87 (77)	87 (77)	87 (79)	86 (75)	86 (75)	86 (75)
**Subgroup V**										
W6 (AF188595)	85 (79)	89 (84)	85 (79)	89 (84)	86 (80)	86 (80)	85 (80)	86 (78)	86 (78)	86 (78)
58P (AF188603)	84 (77)	88 (81)	84 (77)	88(81)	85 (77)	85 (77)	84 (77)	85 (75)	85 (75)	85 (75)
88P (AF188604)	84 (78)	89(82)	84 (78)	89 (82)	85 (78)	85 (78)	84 (78)	85 (76)	85 (76)	85 (76)
P10 (AF188601)	84 (77)	89 (82)	84 (77)	89 (82)	86 (78)	86 (78)	84 (78)	85 (76)	85 (76)	85 (76)
394 (AF188596)	84 (77)	88 (82)	84 (77)	88 (82)	85 (78)	85 (78)	84 (78)	85 (76)	85 (76)	85 (76)
8352 (AF188597)	82 (74)	87 (79)	82 (74)	87 (79)	84 (75)	84 (75)	82 (75)	83 (73)	83 (73)	83 (73)
**Subgroup VI**										
K1 (AF188585)	85 (77)	85 (75)	85 (77)	85 (75)	87 (78)	87 (78)	85 (78)	87 (76)	87 (76)	87 (76)
K2 (AF188586)	85 (77)	85 (75)	85 (77)	85 (75)	87 (78)	87 (78)	85(78)	87 (76)	87 (76)	87 (76)
**Subgroup VII**										
IT24374 (KY753465)	81 (76)	85 (79)	81 (76)	85 (79)	83 (76)	83 (76)	81 (76)	83 (74)	83 (74)	83 (74)
SM56243 (KY753461)	82 (76)	86 (79)	82 (76)	86 (79)	83 (74)	83 (74)	82 (76)	82 (72)	82 (72)	82 (72)
IT22579 (KY753466)	81 (76)	85 (79)	81 (76)	85 (79)	83 (76)	83 (76)	81 (76)	83 (74)	83 (74)	83 (74)
IT11785 (KY753467)	80 (75)	84 (78)	80 (75)	84 (78)	82 (75)	82 (75)	80 (75)	82 (73)	82 (73)	82 (73)
B26/12/CRO (KY680320)	83 (76)	88 (81)	83 (76)	88 (81)	83 (74)	83 (74)	82 (75)	83 (72)	83 (72)	83 (72)
B43(B1)_/11/CRO (KY680325)	83 (76)	87 (81)	83 (76)	87 (81)	83 (74)	83 (74)	82 (75)	82 (72)	82 (72)	82 (72)
B99/11/CRO (KY680328)	82 (76)	86 (80)	82(76)	86 (80)	82 (74)	82 (74)	81 (75)	82 (72)	82 (72)	82 (72)
**Subgroup VIII**										
B60279/2/14/CRO (KY680330)	84 (74)	86 (76)	84 (74)	86 (76)	85 (76)	85 (76)	84 (75)	85 (74)	85 (74)	85 (74)
B59586/1/15/CRO (KY680331)	83 (73)	86 (76)	83 (73)	86 (76)	85 (74)	85 (74)	83 (74)	84 (72)	84 (72)	84 (72)
B10152/3/16/CRO (KY680335)	83 (73)	86 (76)	83(73)	86 (76)	84 (74)	84 (74)	83 (74)	84 (72)	84 (72)	84 (72)
B10152/9/16/CRO (KY680336)	83 (73)	86 (76)	83 (73)	86 (76)	84 (74)	84 (74)	83 (74)	84 (72)	84 (72)	84 (72)
B9156/2/16/CRO (KY680337)	83(73)	86 (76)	83 (73)	86 (76)	84 (74)	84 (74)	83 (74)	84 (72)	84 (72)	84 (72)

**Figure 2.**
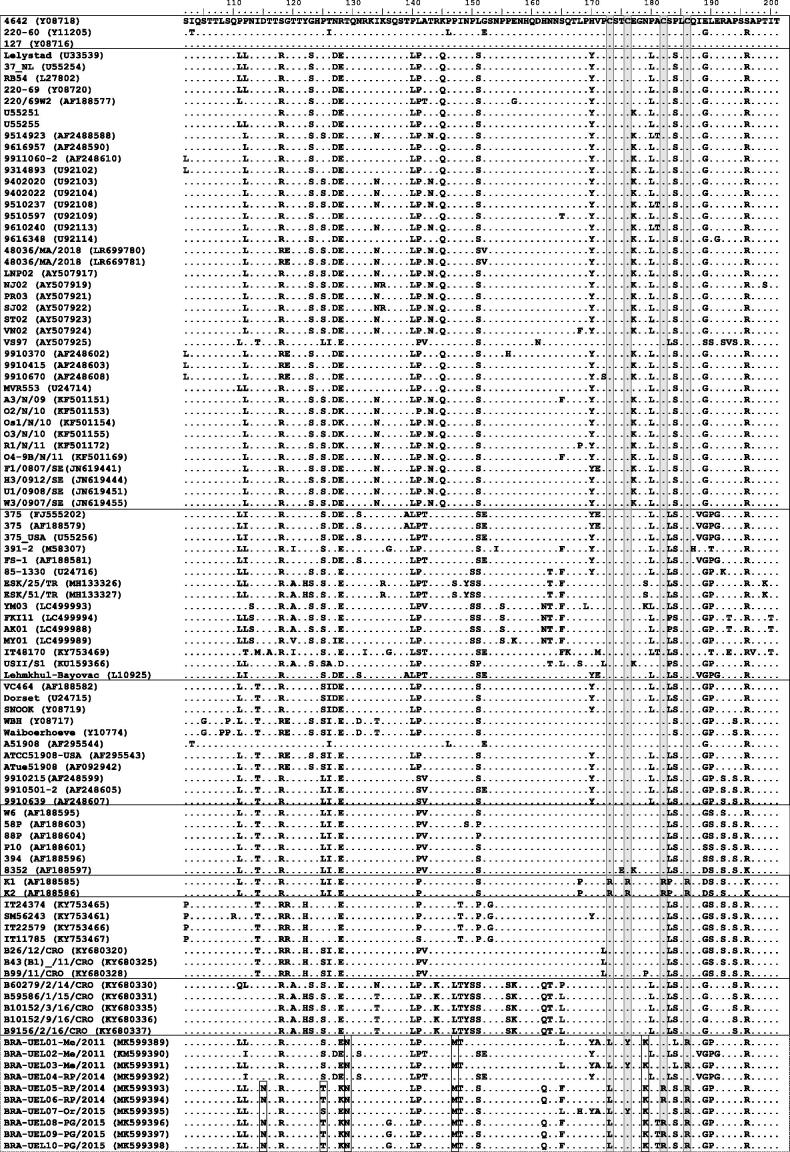
Alignment of the deduced amino acid (aa) sequences of the central hydrophobic region of the G gene of bovine respiratory syncytial virus (BRSV) strains. The aa sequences are boxed according to the subgroups (I to VIII). Brazilian BRSV strains are boxed with a dotted line. Replacements of the conserved cysteine residues are shaded gray. Rectangles show aa changes observed exclusively in the Brazilian strains in this study.

The comparative analysis of the deduced aa sequences from the central hydrophobic region of the G gene among the Brazilian BRSV strains with representative strains showed replacement of the conserved Cys residues at positions 173 (Cys→Leu), 176 (Cys→Tyr), 182 (Cys→Arg), and/or 186 (Cys→Arg). When combined, these replacements were Leu^173^Tyr^176^Cys^182^Arg^186^ for the strains UEL01-Me, 03-Me, and 07-Or, and Leu^173^Cys^176^Arg^182^Arg^186^ for the strains UEL05-RP, 06-RP, 08-PG, 09-PG, and 10-PG. Additionally, the Brazilian BRSV strains also presented substitutions of residues important to antibody reactivity at positions 180 (Leu→Pro), 181 (Ala→Thr), 183 (Leu→Ser), and/or 184 (Ser→Leu). When combined, these replacements were Pro^180^Ala^181^Ser^183^Leu^184^ for the strains UEL01-Me and 03-Me, Pro^180^Ala^181^Ser^183^Ser^184^ for the strains UEL05-RP, 06-RP, and 07-Or, and Pro^180^Ala^181^Ser^183^Ser^184^ for the strains UEL08-PG, 09-PG, and 10-PG. However, no replacement in the cysteine-noose and antibody-binding residues was observed in the UEL02-Me and 04-RP strains. Other aa changes were revealed exclusively in the Brazilian BRSV strains in this study (Figure 2).

**Figure 3.**
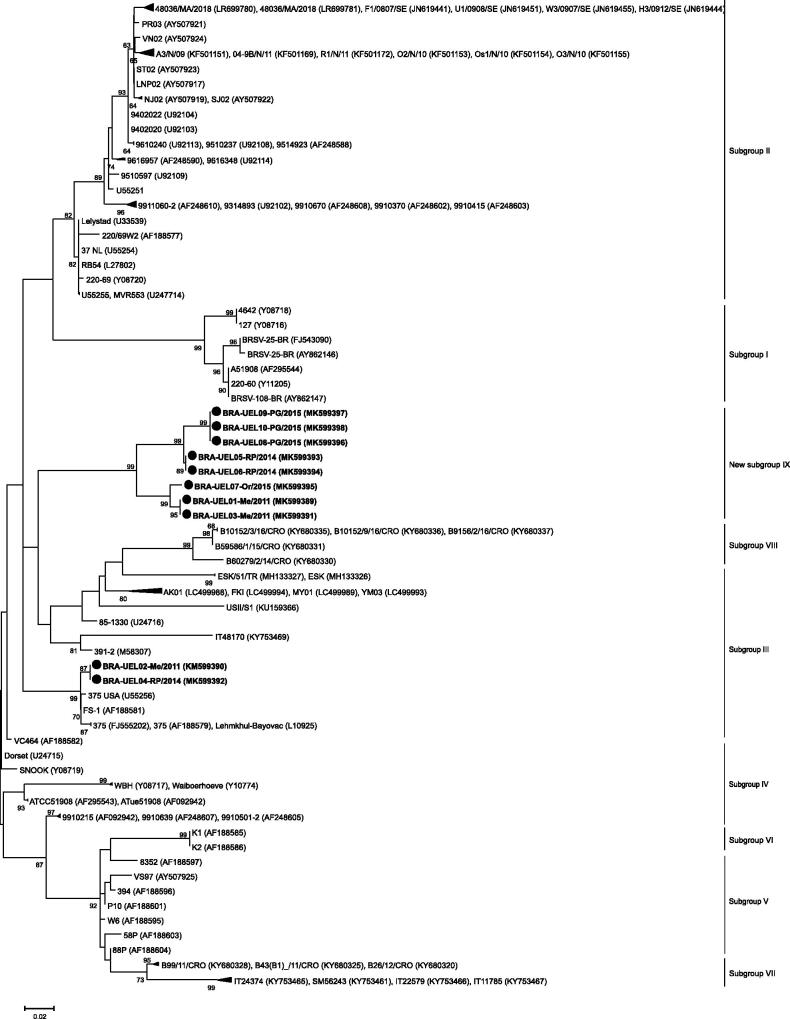
Molecular phylogenetic analysis of the partial (290 nt) G gene of bovine respiratory syncytial virus strains. The evolutionary history was inferred by using the Maximum Likelihood method based on the Tamura-Nei model (Tamura and Nei 1993). A discrete Gamma distribution was used to model evolutionary rate differences among sites (5 categories [+G, parameter = 0.9582]). The analysis involved 102 nucleotide sequences. Bootstrap values higher than 60% are shown. Initial trees for the heuristic search were obtained automatically by applying the Neighbor-joining and BioNJ algorithms to a matrix of pairwise distances estimated using the Maximum Composite Likelihood (MCL) approach and then selecting the topology with a superior log likelihood value. The tree is drawn to scale, with branch lengths measured in the number of substitutions per site. Evolutionary analyses were conducted in MEGA7 (Kumar et al. 2016).

In the phylogenetic analysis of the G gene, the Brazilian strains UEL02-Me and 04-RP grouped together with sequences of subgroup III, the same branch of the Bayovac vaccine strain. The other eight Brazilian strains formed a new branch, distinct from the representative BRSV sequences of I to VIII subgroups (Figure 3).

### 3.2. Gene F analysis

Amplicons with the expected size for the F gene were obtained from all 10 BRSV strains in this study. However, we could not obtain consensus nt sequences with high quality from the strains UEL02-Me, 03-Me, and 08-PG; therefore, these three strains were not included in the analysis.

**Figure 4.**
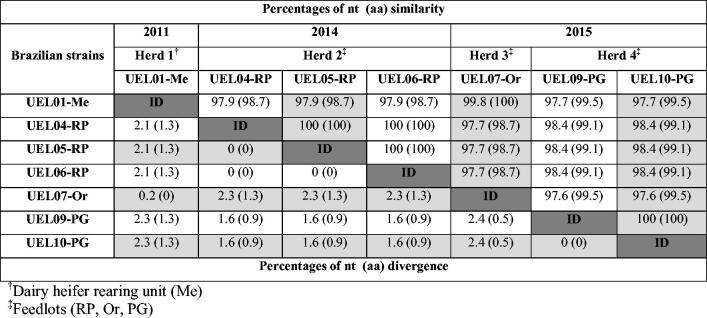
Percentages of similarities and divergences of the nucleotide (nt) and deduced amino acid (aa) sequences among Brazilian bovine respiratory syncytial virus strains based on partial (721 nt) F gene analysis.

Overall, the genetic differences observed in the partial F gene were discrete when compared with those of the G gene. The analysis of the Brazilian BRSV sequences revealed that the partial F genes of the strains UEL04-RP, 05-RP, and 06-RP were 100% identical to each other at both the nt and aa levels. The same could be observed for the UEL09-PG and 10-PG strains. Although there was a slight divergence (0.2%) in nt sequences, the aa sequences of the strains UEL01-Me and 07-Or were 100% identical to each other (Figure 4).

The analysis of the BRSV F gene included representative strains of I to VI subgroups. Comparisons of the Brazilian BRSV nt and aa sequences with the reference sequences showed high similarities, varying from 96 to 98% for nt sequences (98 to 100% for aa sequences) (Table 2).

**Table 2.** Percentages of similarities of the nucleotide and deduced amino acid sequences among the bovine respiratory syncytial virus strains in this study and representative strains of different subgroups based on the partial (721 nt) F gene.

Reference strains (GenBank accession number)	Brazilian strains						
	2011	2014			2015		
	UEL01-Me	UEL04-RP	UEL05-RP	UEL06-RP	UEL07-Or	UEL09-PG	UEL10-PG
**Subgroup I**							
4642 (AF188576)	96 (98)	96 (98)	96 (98)	96 (98)	96 (98)	96 (99)	96 (99)
A51908 (AF295544)	96 (98)	96 (98)	96 (98)	96 (98)	96 (98)	96 (98)	96 (98)
**Subgroup II**							
Lelystad (AF188573)	97 (98)	96 (98)	96 (98)	96 (98)	97 (98)	97 (99)	97 (99)
220-69 (AF188575)	97 (98)	96 (98)	96 (98)	96 (98)	97 (98)	96 (98)	96 (98)
W2 (AF188572)	97 (99)	96 (98)	96 (98)	96 (98)	97 (99)	97 (99)	97 (99)
FV160 (AF188574)	97 (98)	97 (98)	97 (98)	97 (98)	97 (98)	97 (99)	97 (99)
**Subgroup III**							
375 (FJ543092)	98 (99)	98 (98)	98 (98)	98 (98)	98 (99)	98 (99)	98 (99)
391-2 (M58350)	98 (100)	98 (99)	98 (99)	98 (99)	98 (100)	98 (100)	98 (100)
USII/S1 (KU159366)	96 (99)	96 (99)	96 (99)	96 (99)	96 (99)	96 (100)	96 (100)
Bayovac (AF188571)	98 (99)	98 (98)	98 (98)	98 (98)	98 (99)	98 (99)	98 (99)
**Subgroup IV**							
SNOOK (Y17970)	98 (99)	97 (99)	97 (99)	97 (99)	98 (99)	97 (100)	97 (100)
5761 (AF188570)	98 (99)	97 (99)	97 (99)	97 (99)	97 (99)	97 (100)	97 (100)
90504 (AF188562)	98 (100)	97 (99)	97 (99)	97 (99)	98 (100)	97 (100)	97 (100)
V347 (AF188569)	98 (100)	97 (99)	97 (99)	97 (99)	98 (100)	97 (100)	97 (100)
ATCC51908 (AF295543)	98 (99)	97 (98)	97 (98)	97 (98)	98 (99)	97 (99)	97 (99)
ATue51908 (AF092942)	98 (99)	97 (98)	97 (98)	97 (98)	98 (99)	97 (99)	97 (99)
ATCC51908 (NC_038272)	98 (99)	97 (98)	97 (98)	97 (98)	98 (99)	97 (99)	97 (99)
**Subgroup V**							
W6 (AF188565)	98 (100)	97 (99)	97 (99)	97 (99)	98 (100)	97 (100)	97 (100)
A1 (AF188555)	98 (100)	97 (99)	97 (99)	97 (99)	97 (100)	97 (100)	97 (100)
A2Gelfi (AH008996)	98 (100)	97 (99)	97 (99)	97 (99)	97 (100)	97 (100)	97 (100)
B1 (AF188556)	98 (100)	97 (99)	97 (99)	97 (99)	97 (100)	97 (100)	97 (100)
B2 (AF188557)	98 (100)	97 (99)	97 (99)	97 (99)	97 (100)	97 (100)	97 (100)
C2 (AF188558)	98 (100)	97 (99)	97 (99)	97 (99)	97 (100)	97 (100)	97 (100)
394 (AF188554)	98 (100)	97 (99)	97 (99)	97 (99)	97 (100)	97 (100)	97 (100)
F2 (AF188564)	97 (99)	97 (99)	97 (99)	97 (99)	97 (99)	97 (100)	97 (100)
G2 (AF188559)	98 (100)	97(99)	97 (99)	97 (99)	97(100)	97 (100)	97 (100)
J1 (AF188560)	97 (100)	97 (99)	97 (99)	97 (99)	97 (100)	97 (100)	97 (100)
88P (AF188568)	97 (99)	97 (99)	97 (99)	97 (99)	97 (99)	97(100)	97 (100)
L1 (AF188563)	97 (100)	97 (99)	97 (99)	97 (99)	97 (100)	97 (100)	97 (100)
P3 (AF188566)	98 (100)	97 (99)	97 (99)	97 (99)	98 (100)	97 (100)	97 (100)
P8 (AF188567)	97 (100)	97 (99)	97(99)	97 (99)	97 (100)	97 (100)	97 (100)
**Subgroup VI**							
K1 (AF188561)	97 (100)	97 (99)	97 (99)	97 (99)	97 (100)	97 (100)	97 (100)

**Figure 5.**
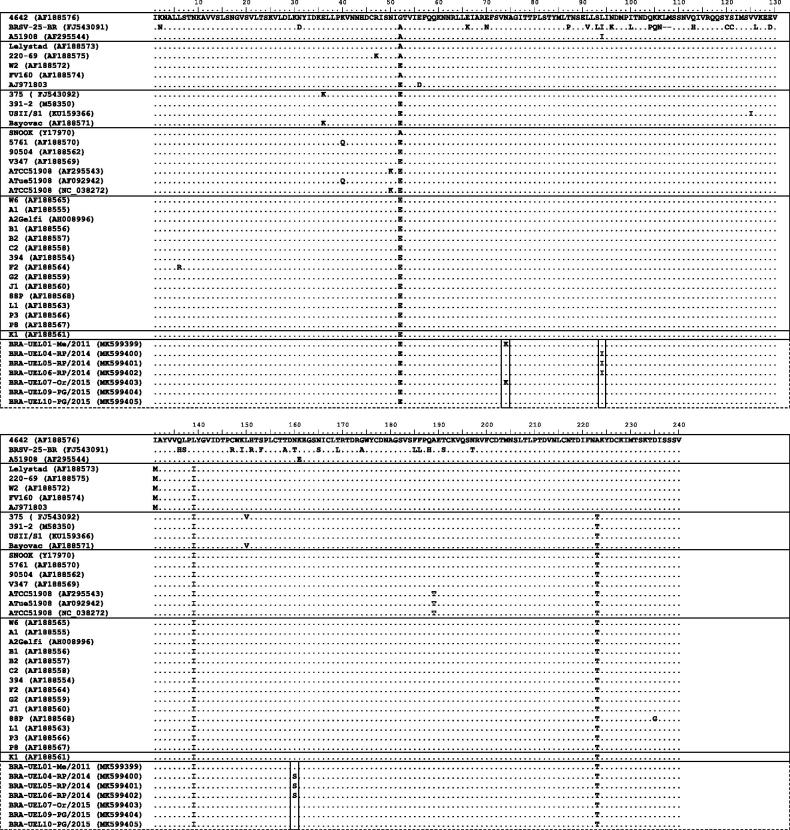
Alignment of the deduced amino acid (aa) sequences of the F gene of bovine respiratory syncytial virus (BRSV) strains. The aa sequences are boxed according to the subgroups (I to VI). The Brazilian BRSV strains are boxed with a dotted line. Rectangles show aa changes observed exclusively in the Brazilian strains in this study.

**Figure 6.**
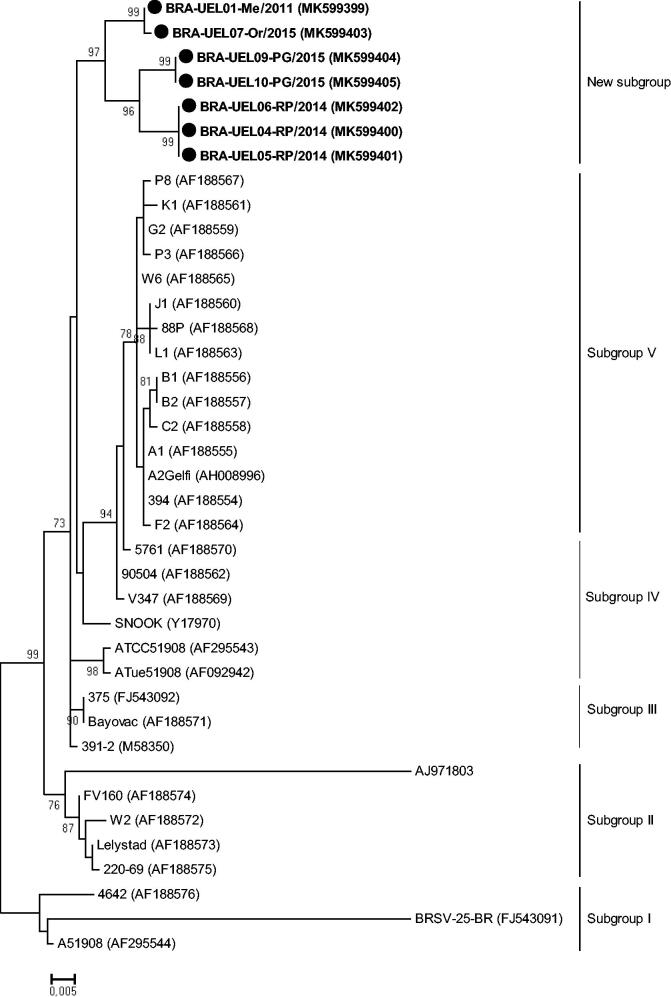
Molecular phylogenetic analysis of the partial (715 nt) F gene of bovine respiratory syncytial virus. The evolutionary history was inferred by using the Maximum Likelihood method based on the Tamura 3-parameter model (Tamura 1992). A discrete Gamma distribution was used to model evolutionary rate differences among sites (5 categories [+G, parameter = 0.2848]). The analysis involved 39 nucleotide sequences. Bootstrap values higher than 60% are shown. Initial tree(s) for the heuristic search were obtained automatically by applying Neighbor-Joining and BioNJ algorithms to a matrix of pairwise distances estimated using the Maximum Composite Likelihood (MCL) approach and then selecting the topology with a superior log likelihood value. The tree is drawn to scale, with branch lengths measured in the number of substitutions per site. Evolutionary analyses were conducted in MEGA7 (Kumar et al. 2016).

Although highly conserved, the deduced aa sequences from the Brazilian BRSV strains showed aa changes that were not present in the representative sequences of the I to VI subgroups (Figure 5). Additionally, the phylogenetic analysis showed that the Brazilian BRSV strains grouped in a branch different from the other representative strains (Figure 6).

### 3.3. Viral isolation

Viral isolation on the nasopharyngeal swab suspensions was performed but failed to isolate any virus, including BRSV.

## 4. Discussion

The available molecular data on Brazilian wild-type strains are limited. This study aimed to molecularly characterize BRSV strains identified in BRD-affected dairy heifers and steers in feedlots. Brazilian BRSV sequences of both the F and G genes showed unique aa changes, including within the immunodominant region of the G protein. The exceptions were the strains UEL02-Me/2011 and 04-RP/2014, which were classified as belonging to subgroup III, with high similarity to the Bayovac vaccine strain. Since these strains are highly conserved, they will not be discussed in depth.

The partial F gene analysis showed slight differences among the Brazilian strains (as much as 2.4% higher and 1.3% higher at the nt and aa levels, respectively) and among the strains representative of each known subgroup (4.5% and 2.5% at the nt and aa levels, respectively). However, we could not perform in-depth analysis of the F gene in this study since the partial amplified sequences were not targeted to important domains of this gene, such as glycosylation sites and/or hydrophobic motifs. Although highly conserved, the detailed comparison of the sequences from the partial F gene of the BRSV strains in this study showed exclusive aa changes in relation to the prototype strains. Interestingly, the comparison of the BRSV strains with another previously described Brazilian strain revealed higher divergences (10.9% and 16.7% at the nt and aa levels, respectively). These results reveal the genetic diversity of the Brazilian BRSV strains, even from a conserved genomic region within the F gene of the virus.

The partial G gene analysis showed that the Brazilian strains identified in this study are different from the Bayovac vaccine and other strains of the I to VIII subgroups, with nt divergences varying from 10.7 to 15.5% (11 to 26% at the aa level). In addition, previous BRSV strains derived from Brazilian cattle herds were shown to be genetically related to subgroup I (Domingues et al. 2011). However, when compared with the BRSV sequences in this study, the nt divergences ranged from 15 to 17.3% (26 to 30% at the aa level). Although cut-off values at both the nt and aa levels are not currently established and considering that the nt differences observed in the BRSV prototype strains among each other vary from 5% to 16% (Sarmiento-Silva et al. 2012), it is highly likely that the Brazilian strains in this study represent a putative new subgroup of BRSV. Furthermore, both G and F gene-based phylogenetic analyses showed major branches consisting of strains representative of each subgroup. However, a different branch in each tree held only the Brazilian strains identified in this study, reinforcing the hypothesis that these are representative of a new BRSV subgroup, tentatively named subgroup IX.

Phylogenetic analyses of both the G and F genes also showed that the branches including the Brazilian BRSV strains were further subdivided into clusters according to the herd of origin. The G protein, when compared with the other BRSV proteins, such as F and N, has a higher rate of genetic variability (Valarcher et al. 2000). Usually, it is possible to observe a very limited difference (Valarcher et al. 2000) or complete similarity between the BRSV strains found in animals of the same herd (Larsen 2000; Larsen et al. 2000), revealing that a single virus or group of closely related viruses predominantly infect a given herd at a given time (Valarcher et al. 2000). However, the UEL04-RP and UEL05-06RP strains, which were derived from the same feedlot and collected at the same time from BRD-affected steers, were 13.3% divergent at the nt level (23% at the aa level) between each other and grouped in different clusters. The UEL01-Me and 03-Me strains, also collected at the same time in the same dairy heifer rearing unit, were 100% identical to each other; however, when compared with the strain UEL02-Me, they demonstrated lower nt and aa (87% and 79%, respectively) similarity. These findings suggest that there are different lineages of BRSV circulating in Paraná state and even in a single herd at the same time.

All Brazilian BRSV strains in this study showed aa changes within the ectodomain region of the G gene, including in the G-central conserved region between aas 158-189. We expected to find a conserved hydrophobic domain between residues 173-186; however, substitutions of cysteines and other residues that have a great influence on immunological activity were observed. Additionally, the Brazilian BRSV strains lacked both the Asn^179^ and Leu^183^ residues, which were replaced by Lys and Ser, respectively. Cysteine residues are important factors for the intramolecular disulfide bond and steric structure of the protein. Cys^173^-Cys^186^ and Cys^176^–Cys^182^ constitute the outer and inner disulfide bridges, respectively, while Asn^179^ is involved in three hydrogen bonds that link the two helices of the Cys^176^-disulfide bridge via backbone oxygen with the Leu^183^ amide in the second helix (Doreleijers et al. 1996). In addition, although the cysteine noose was shown to be unnecessary for effective virus infection both *in vitro* and *in vivo* (Teng and Collins 2002), it was considered necessary for binding the virus to the cell (Akerlind-Stopner et al. 1990, Valentova et al. 2012). Therefore, it is likely that the lack of the three cysteines that were replaced by Leu, Tyr, and/or Arg and other residue mutations at positions 179 and 183 observed in the Brazilian BRSV strains in this study represent a loss of disulfide bridges and structural changes in the G protein.

Conversely, the replacement of all Cys residues with Arg, resulting in changes in the structure of the central conserved region and the disturbance of the α helix Cys^173^-Cys^176^, was previously reported in BRSV strains isolated from diseased animals (Valarcher et al. 2000). This demonstrated that natural *in vivo* infections with mutants lacking the four cysteines involved in the two disulfide bridges are possible. In contrast to previous Brazilian studies (Baptista et al. 2017, Headley et al. 2017, 2018), the nasopharyngeal swabs from which these BRSV strains were obtained were negative for other respiratory tract pathogens, suggesting that these Brazilian BRSV strains might have induced the disease. Additionally, BRSV strains in this study were not isolated in cell culture, suggesting that these may be attenuated strains of the virus. Despite the disulfide bridge loss and structural changes in the G protein, it is likely that the pathogenic potential of these BRSV strains was not affected regardless of whether the strains were attenuated. Although studies have shown that the F protein alone is sufficient to mediate attachment and fusion in the absence of G protein (Valarcher et al. 2007), further studies on these Brazilian BRSV strains should be performed to establish their pathogenic potential.

In this regard, we presume that the potential pathogenic role of some of the Brazilian strains may also have been impacted by mutations at residue 171. A characteristic hydrophobic pocket was previously reported from this genomic region, lined only by the conserved residues Val^171^-Pro^172^, Cys^173^, Cys^176^ and Cys^182^, and Leu^185^-Cys^186^, suggesting that there may be a hydrophobic interaction between the conserved pocket and receptor site that is common to all RSV-G cell receptors (Doreleijers et al. 1996). In this study, along with cysteines, three of the Brazilian BRSV strains were also found with substitutions of the residue Val^171^→Ala^171^, likely influencing the previously mentioned interaction.

Furthermore, studies have reported that residues at positions 174-185 of the central conserved region are immunodominant, and one-point substitutions in this region, especially residues 180, 183, and 184, substantially influence the antibody reactivity of some BRSV strains (Furze et al. 1997, Langedijk et al. 1997). According to linear epitopes, the point mutations that determine BRSV subgroups are Leu^180^ for subgroups A and intermediate and Pro^180^, Ser^183^, and Pro^184^ for subgroup B (Langedijk et al. 1997). Substitutions of these residues with Leu^180^→Pro^180^, Leu^183^→Ser^183^, and/or Ser^184^→Leu^184^ were found in the Brazilian BRSV strains in this study. Moreover, an additional mutation, Ala^181^→Thr^181^, was also observed in three of these strains. When combined, the expected motifs LACLS (subgroup A), PACSP (subgroup B), and LACSS (intermediate subgroup) at position 180-184 were replaced by different residue combinations, such as PACSL, PARSS, PACSS, PTRSS, and PARSS. Although the antigenic reactivity of the Brazilian BRSV strains in this study has not been analyzed, we presume that these strains present changes in antigenic epitopes that also may influence antibody reactivity and binding. Additional studies based on antigenic properties of these Brazilian BRSV strains should be performed to confirm these hypotheses.

A feature of BRSV is its ability to infect a host even in the presence of virus-neutralizing antibodies and to induce reinfections throughout the life of a host (Valentova 2012). Massive vaccination of herds or constant reinfection of animals that already have antibodies against BRSV, as reported in infections by HRSV, were assumed to be important immunologic pressure factors that lead to the expression of virus evolution (Martinez et al. 1997, Prozzi et al. 1997, Valarcher et al. 2000). In this study, the sampled Brazilian cattle herds were not subjected to any direct vaccine selective pressure, and the nt and aa sequences were obtained directly from biological samples of BRD-affected animals, representing wild-type Brazilian BRSV strains with no previous adaption and passages in cell culture. Although BRSV vaccination has been more widely used in Brazil, such vaccination prevents the disease but does not suppress virus circulation (Valarcher et al. 2000). Therefore, the possibility that an indirect vaccination pressure or constant BRSV reinfection has driven mutations in the BRSV strains analyzed in this study cannot be ruled out.

In summary, Brazilian BRSV sequences from both F and G genes showed unique aa changes, including within the immunodominant region of the G protein. Phylogenetic analyses showed that the Brazilian BRSV strains grouped into distinct branches for both genes analyzed, revealing that these represent a putative new subgroup of BRSV. The mutations observed in the Brazilian BRSV strains suggest changes in the structure of the G protein. Considering that these BRSV strains were obtained from BRD-affected animals and based on i) the circulation of distinct lineages of BRSV strains in a single herd at the same time, ii) the changes in the immunodominant region of G protein, and iii) the negative results for the other respiratory tract pathogens, we presume that infection with related but distinct BRSV strains may favor immune evasion by the virus, establishment of infection, and eventually, disease induction, even if these strains are or are not attenuated strains.

In conclusion, considering the genetic variability of BRSV, molecular and epidemiological studies are needed to assess the practical implications of this diversity regarding both the pathogenicity of virus strains and the immunoprophylactic aspects of infection. Changes in the BRSV G protein may even confer cross-protective immunity in animals infected with heterologous strains; however, some aspects such as the degree of protection, scope, and duration of immunity conferred by different subgroups are not yet fully known. The identification and characterization of genetically different BRSV subgroups circulating in beef and dairy cattle herds and further studies on their antigenic properties are critical to the adoption of effective strategies for the control and prophylaxis of BRD and to minimize economic losses. Only with the knowledge of classical and molecular epidemiology of BRSV in a region and/or in a country and the temporal distribution is it possible to implement and evaluate the efficiency of immunoprophylactic programs against BRSV infection in cattle herds around the world.

## Supplementary Material

Supplemental Material

## Data Availability

GenBank accession numbers of BRSV strains herein are MK599389 to MK599398 to the G gene and MK599399 to MK599405 to the F gene.
